# Analysis of serological profiles in SPF chickens infected with classical or variant infectious bronchitis viruses

**DOI:** 10.1016/j.psj.2026.107074

**Published:** 2026-05-05

**Authors:** Sirorat Munyahongse, Nicholas J. Evans, Kannan Ganapathy

**Affiliations:** a Institute of Infection, Veterinary and Ecological Sciences, University of Liverpool, Leahurst Campus, Cheshire, UK; b Department of Science, Technology and Innovation, Faculty of Science, Chulabhorn Royal Academy, Bangkok, Thailand

**Keywords:** Chicken, IBV, ELISA, HI, Serology

## Abstract

Infectious bronchitis virus (IBV) remains a major challenge in poultry farming. Both an ELISA assay and Haemagglutination Inhibition (HI) test are standard serological methods for detecting antibodies against IBV, to monitor vaccine efficacy or to evaluate potential exposure to virulent field strains. This study evaluates clinical and serological differences in SPF chicks following infection with IBV strains QX, M41 or 793B. Clinically, the onset of respiratory signs varied among strains. All three IBV strains (QX, M41, and 793B) caused respiratory signs, with the M41-inoculated birds exhibiting the highest number of respiratory signs. For the detection of IBV antibodies, significant differences were observed among ELISA kits. The QX antisera group exhibited the highest correlation between ELISA kits, whereas the 793B group showed the lowest. Homologous HI titres correlated strongly with ELISA kits A, B, and C in the QX antisera group, whereas other homologous HI titres showed no significant correlation with any ELISA kit. Heterologous HI tests showed high sensitivity, demonstrating some antigenic similarity among QX, M41, and 793B. However, reduced cross-reactivity indicates that this assay can identify the dominant infection strain in flocks. ELISA kit A was the most effective for QX antisera, and other kits showed variable sensitivity. The performance of the ELISA kit varied depending on the specific kit and the antisera group. Principal component analysis (PCA) identified two major components explaining 67.52% of the variance, capturing ELISA and HI test variability. Strong correlations were observed between ELISA kits A and B, as evidenced by Pearson correlation coefficients. Additionally, the M41 HI titre predicted the positivity of the ELISA kits B and C, indicating that false-negative ELISA results were observed when the M41 HI titres were low. Our findings emphasise the need to select the appropriate serological method for each antisera group to ensure accurate detection of different IBV strains.

## Introduction

Serology is extensively utilised in poultry farming for disease diagnosis (Comin et al., 2013), immunological monitoring (Takase et al., 2000), and vaccine efficacy evaluation (Chhabra et al., 2015). These tests are crucial for maintaining health and productivity of poultry (Liebhart et al., 2023). Among serology tests for infectious bronchitis virus (IBV) antibody determination, the haemagglutination inhibition (HI) test, the serum neutralisation test, and ELISA are the most prominent (Villarreal, 2010). IBV serotypes can be identified using the HI and neutralisation tests, with the latter being the gold standard for differentiating between serotypes (de Wit et al., 2019). However, this method is not routinely employed due to the necessity for specific strains and antisera owing to antigenic diversity (Hitchner, 1973). Additionally, it demands specialised techniques and conditions because of its narrow cell tropism (Bickerton et al., 2018), and it has limitations in classifying new strains (Lee et al., 2001). The HI test is frequently used to identify serotype-specific responses to vaccination in young birds and for diagnostic purposes (World Organisation for Animal Health (WOAH), 2018). However, its sensitivity and specificity can be affected by limited cross-reactivity among strains (Lashgari and Newman, 1984b). ELISA, developed for measuring antibody responses, is more popular as it provides high accuracy, sensitivity, and convenience (Ji et al., 2022). It enables the detection and quantification of specific antibodies, making it an invaluable tool in IBV research and diagnostic settings (Marquardt et al., 1981). Molecular techniques are also employed for strain classification (Lin and Chen, 2017). Variability in ELISA kit results stems from differences in coating antigens and approaches, as well as other standardisation protocols, such as reagents and incubation conditions, leading to varied antibody detection efficiency and interpretations of results (Alhajj et al., 2023). Although commercial ELISA kits for IBV are available, comparative performance evaluations of the kits using antisera produced against different IBV strains are limited. Antiserum contains specific antibodies that are advantageous for both human and animal research (Munoz and Becker, 1952), including diagnostic tools (Pillai-Kastoori et al., 2020) and treatment regimens (Mohawk et al., 2010).

Antiserum offers several benefits for IBV serology tests in poultry, including standardisation and validation of the test results (Perrotta et al., 1988). It facilitates the identification of Mycoplasma strains through an indirect fluorescent antibody test (Bradbury, 1982) and the determination of avian virus subtypes by the HI test, such as avian influenza virus (Lee et al., 2006) and Newcastle disease virus (Mao et al., 2022). Antiserum additionally neutralises the oocytes of Eimeria (Herlich, 1965). As a primary antibody that targets specific vaccine antigens or proteins, antisera are an important diagnostic tool in nucleic acid vaccine studies (Sun et al., 2005).

This study reports the detection of IBV antibodies in antisera produced against three different IBV genotypes using various commercial ELISA kits. The performance of three commercial ELISA kits was evaluated and compared with homologous and heterologous HI tests. Additionally, the study includes a comparative analysis of the severity and duration of clinical signs following inoculation with IBV strains M41, 793B, and QX.

## Materials and methods

### Ethical statement

All procedures were approved under project licence number P8E4FC2C9 (Protocol No. 5: Raising Polyclonal Antisera) by the University of Liverpool Ethical Review Committee. Chicks were provided feed and water *ad libitum* and raised according to animal welfare guidelines under strict biosecurity measures at the University of Liverpool. All birds were humanely euthanised by overdose injection of Pentobarbital (20% w/v Solution for injection, 0.7 ml/kg) in accordance with the UK Legislation: Animals (Scientific Procedures) Act 1986, Schedule 1.

### Challenge virus

The third passage of M41, the fifth passage of 793B, and QX IBV-rich allantoic were propagated in the SPF embryonated chicken eggs and harvested as stock viruses. All three IBV viruses were ruled out for other avian viral pathogens by PCR (Aldous et al., 2003; Beltrão et al., 2012; Cavanagh et al., 1999; Mase and Kanehira, 2015), and bacterial and fungal contamination by culture. They were then titrated into tracheal organ cultures (TOCs) and SPF embryonated chicken eggs to determine the virus titre, given 10^7.5^, 10^7.32^, and 10^7.17^ CD_50_/ml, and 10^7.7^, 10^6.33^, and 10^6.7^ EID_50_/ml, respectively.

### Animal studies

Embryonated SPF eggs of White Leghorn chickens were incubated at 37°C for 21 days. Sixty-one chicks were then equally divided into four groups, including QX (n = 16), M41 (n = 15), 793B (n = 15), and the non-challenge group (n = 15). One hundred microliters of IBV-rich allantoic fluid of each strain at 10^6.5,^ 10^6.32^, and 10^6.17^ CD_50_/ bird were inoculated oculo-nasally at three weeks old to QX, M41, and 793B groups, respectively. The non-challenge group (NC) was given the same pathogen-free allantoic fluid. Seven days after the initial challenge, respective groups of birds were inoculated with the same strain via the same route of administration. Respiratory and clinical signs associated with IBV infection, including snicking, headshaking and scratching, were recorded twice daily. All birds were humanely culled three weeks after the second challenge, and blood samples were individually collected. Separated sera were inactivated at 56°C and stored at −20°C until use.

### Serology tests

Serum IBV antibody titres were assayed using two methods, HI and ELISA. Homologous and heterologous HI tests were performed using QX (D388), M41, and 793B antigen by Sci-Tech Laboratory (Cawood, UK). The HI titre was expressed as a log_2_ transformation. For ELISA, three different commercial ELISA kits were used according to the manufacturer's instructions. Briefly, sera were diluted 1:500 for kits A, B, and C, and each was tested in triplicate. Thereafter, conjugate and substrate were added, and absorbance was recorded at an appropriate wavelength corresponding to the manufacturer's substrate. ELISA results were reported as S/P ratio or percentage inhibition as follows:

$$\frac{S}{P}ratio=\;\frac{OD\;of\;sample}{OD\;of\;positive\;control}$$

### Mathematical approach for serological test results analysis

**Sensitivity test.** Each ELISA kit and HI test was measured for sensitivity, which measures the serological test's ability to detect the presence of antibodies (Chen et al., 2011). The sensitivity test was performed using the following formula:

$$Sensitivity=\frac{\text{True}\;\text{positive}\;\left(\text{TP}\right)}{\text{True}\;\text{positive}\;\left(\text{TP}\right)+\text{False}\;\text{negative}\;\left(\text{FN}\right)}$$

**Percentage of antigenic relationship.** The percentage of antigenic relationship was measured to quantify antigenic relatedness between two IBV antigens. The estimated antigenic relationships of QX, M41, and 793B strains were calculated following the equation as previously described (Lashgari and Newman, 1984b).

$$r1\;or\;r2=\frac{\text{HI}\;\text{activity}\;\text{of}\;\text{serum}\;\text{against}\;\text{heterologous}\;\text{strain}}{\text{HI}\;\text{activity}\;\text{of}\;\text{serum}\;\text{against}\;\text{homologous}\;\text{strain}}$$

$$Percentage\;of\;antigenic\;relationships=\;100\;x\;\sqrt{r1xr2}$$

Where: r1 represents the relative HI titres of the heterologous antigen, r2 represents the relative HI titres of the homologous antigen

**Cross-reactivity index (CRI).** The cross-reactivity index was assessed to evaluate antibodies induced by one IBV strain against heterologous strains, as previously reported (Lashgari and Newman, 1984a).

$$Cross-reactivity\;index=\frac{\text{Mean}\;log_{2}\;\text{heterologous}\;\text{HI}\;\text{titre}}{\text{Mean}\;log_{2}\;\text{homologous}\;\text{HI}\;\text{titre}}$$

### Statistical analysis

Statistical analyses were performed to evaluate the performance and interrelationships of various serology tests. Statistical analysis and graphs were generated using GraphPad Prism 10.1.2 for Windows (GraphPad Software, USA). An asterisk denoted the significant levels (*), where ****, ***, ** and * indicate a p-value of < 0.0001, < 0.001, 0.01 and < 0.05, respectively. Two-way ANOVA: A two-way ANOVA was performed to compare geometric mean values and assess variations across three antisera groups, considering the influence of both serology tests and antisera group. Post-hoc multiple comparisons were conducted using Tukey’s test to determine statistically significant differences among group means.

**Pearson correlation coefficients.** Pearson correlation coefficients with corresponding 95% confidence intervals were calculated to evaluate the strength and direction of linear relationships between serology test values. Correlation coefficients (r) were interpreted according to conventional strength thresholds, where r near 1 reflects strong relationships, whereas values near 0 indicate weak or no linear association. A positive correlation indicates that both test values increase together, whereas a negative correlation indicates that one increases as the other decreases.

**Simple linear regression analysis.** Simple linear regression was employed to model the relationship between two test values and estimate the expected value of one assay based on the test values of the other. This method enables predictive analysis by quantifying how changes in one test value correspond to changes in the other. The prediction was expressed using the following linear equation:

Y=a+bX

Where:

Y represents the predicted test value,

X represents the predictor test value, a represents the intercept, b represents the slope of the regression line

**Principal component analysis (PCA).** PCA was applied to reduce data dimensionality and explore underlying patterns within the dataset, including serology test values and antisera groups. This multivariate technique transforms correlated variables into a set of uncorrelated components, shown as principal components (PCs), which capture the maximum variance in the data. PCA facilitated the identification of correlations among tests and allowed for detecting deviations along the PCs, adding to visualisation and interpretation of test variability. In the PCA loading plot, direction and angle of each serology test in the PCA loadings indicate the degree of correlation between tests. In comparison, the length of the arrows reflects the strength of each test’s influence in defining the PCs. The PCA score plot illustrates the distinction between antisera groups based on antibody profiles. In addition, the combined interpretation of the score and loading plots provides insights into which assays may be more informative for characterising the serological patterns of each antisera group.

**Logistic regression.** To further evaluate the relationship between test results, logistic regression was performed using HI titres as continuous predictors and ELISA test outcomes as a binary variable (positive vs. negative). This analysis assessed whether variations in HI titres were significantly associated with ELISA positivity.

## Results

### Comparative clinical signs of three IBV strains

**Fig. 1:**
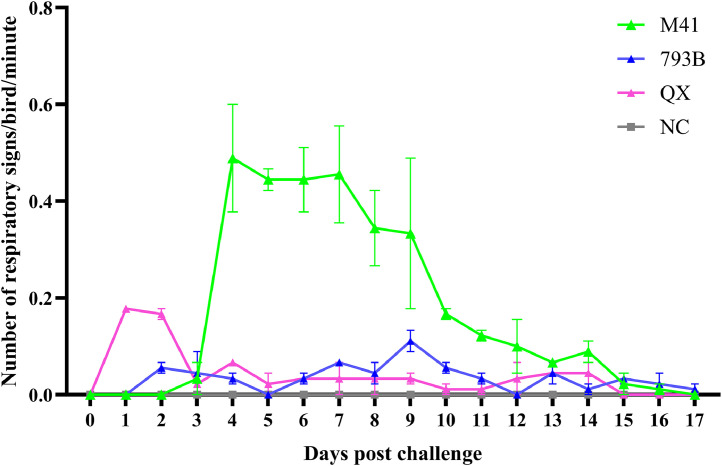
The number of IBV-related respiratory signs (snicking, headshaking and scratching) per bird per minute was recorded twice daily. The M41 antisera, 793B antisera, and QX antisera groups represent the M41 IBV, 793B, and QX inoculated groups, respectively. The NC group represents the pathogen-free allantoic fluid inoculated group. The number of IBV-related respiratory signs is shown as the mean, with the error bar indicating the standard error of the mean.

Prior to the challenge, all groups remained free of clinical signs. Following the first M41 inoculation, birds exhibited IBV-related clinical signs from day 3 until day 16 post-challenge. The signs include wheezing, snicking, headshaking, scratching, tracheal rales, and mild dullness. The number of clinical signs in the M41- and QX-inoculated groups peaked on day 4 and 1 post-first inoculation, respectively. Meanwhile, the 793B inoculated group showed the highest respiratory signs at nine days post-inoculation. The M41 inoculated group exhibited clinical signs at 3 days post-challenge. In contrast, the QX and 793B groups showed clinical signs on days 1 and 2 post-challenge, respectively (Fig. 1). The M41 group showed the highest number of respiratory signs compared to the other strains. In addition to respiratory signs, one bird in the 793B group showed signs of depression and ruffled feathers. In the QX-inoculated group, two birds exhibited gasping during the first three days post-inoculation. No clinical signs were observed in the control group throughout the experiment.

### Homologous and heterologous cross-reactivity among three strains of antisera

HI test results demonstrated 100% sensitivity for all homologous HI, as all samples tested positive (Table 1). In the heterologous HI, a few samples (one out of 15 or 16) showed negative results (below the cut-off value). The calculated sensitivity of the heterologous HI test was 93.3%, 93.8% and 93.3% for antisera of 793B against QX, QX against M41, and M41 against 793B HI test, respectively (Table 1).

**Table 1:** The sensitivity and specificity of 3 commercial ELISA kits and HI test against different IBV antigens compared to the homologous HI test.

	Antisera	ELISA kits			HI tests		
		A	B	C	QX HI	M41 HI	793B HI
**Sensitivity**	QX	87.5^a^ (14^b^/16^c^)	68.8 (11/16)	31.3 (5/16)	100 (16/16)	93.8 (15/16)	100.0 (16/16)
	M41	93.3 (14/15)	100.0 (15/15)	86.7 (13/15)	100.0 (15/15)	100 (15/15)	93.3 (14/15)
	793B	100.0 (15/15)	100.0 (15/15)	73.3 (11/15)	93.3 (14/15)	100.0 (15/15)	100 (15/15)
**Specificity**	QX	100.0^d^	100.0	100.0	100.0	100.0	100.0
	M41	100.0	100.0	100.0	100.0	100.0	100.0
	793B	100.0	100.0	100.0	100.0	100.0	100.0

a Sensitivity.

b Number of positive.

c Number of samples.

d Specificity.

**Fig. 2:**
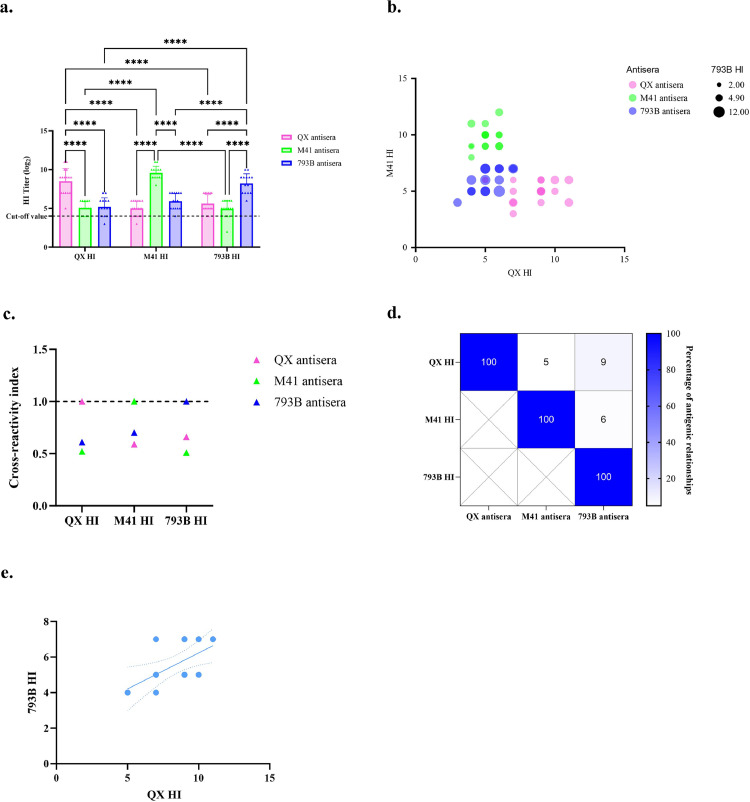
Homologous and heterologous cross-reactivity among three strains of antisera. Mean HI titre (log2) comparing homologous and heterologous HI tests using Two-way ANOVA (a). Bubble plot of heterologous and homologous HI titre (b). Cross-reactivity index of 3 antisera groups (c). Percentage of antigenic relationships of three IBV antigens (d). Simple linear regression of 793B HI and QX HI titre of QX antisera (e) with R-squared, p-value, and equation are 0.3484, 0.0161, and y = 0.4048x + 2.185, respectively. An asterisk denoted the significant levels (*), where ****, ***, ** and * indicate a p-value of < 0.0001, < 0.001, 0.01 and < 0.05, respectively.

The homologous HI titres were significantly higher than the heterologous HI titres and markedly reduced across all heterologous antisera groups (Fig. 2a). The negative results were observed in the following HI titres: 2^3^: 793B antisera with QX HI, 2^3^: QX antisera with M41-HI, and 2^2^: M41 antisera with 793B HI (Fig. 2a). The bubble plot summarises the full dataset of homologous and heterologous HI titres, providing an overview of antibody responses across all tested strains (Fig. 2b). The estimation of antigenic relationships among three antisera groups is expressed as the cross-reactivity index, and the estimation of the relationship between strains. The cross-reactivity index of homologous HI tests was 1, but heterologous HI tests ranged from 0.51 to 0.7. The QX antigen showed cross-reactivity indices of 0.52 and 0.61 against M41 and 793B antisera, respectively (Fig. 2c). The M41 antigen exhibited cross-reactivity indices of 0.59 and 0.70 against QX and 793B antisera, respectively. The 793B antigen showed cross-reactivity indices of 0.66 and 0.51 against QX and M41 antisera, respectively. The estimation of the relationships between homologous antigens was 100%, whereas the relationships between QX and M41, QX and 793B, and M41 and 793B were 5%, 9%, and 6%, respectively. There was no significant correlation among heterologous HI tests, except for QX HI and 793B HI of QX antisera, as determined by Pearson correlation (Fig. 2d). The simple linear regression analysis revealed a significant positive relationship between the QX HI titre and the 793B HI titre of QX antisera, with an R-squared value of 0.3484, a p-value of 0.0161, and the equation y = 0.4048x + 2.185 (Fig. 2e).

### Comparison of the performance of different commercial kits using different IBV antisera

**Fig. 3:**
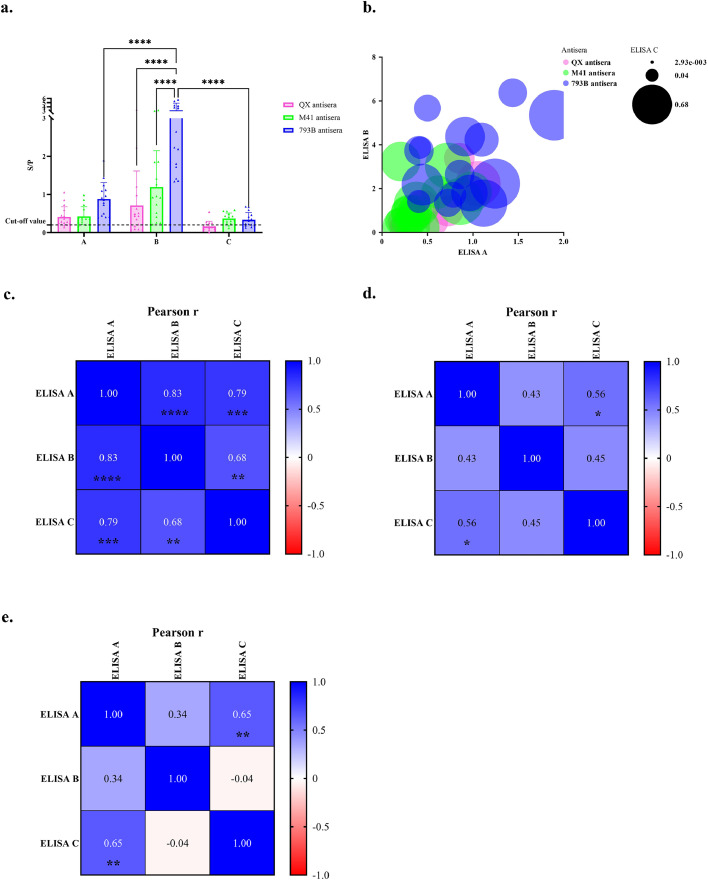
Comparison of the performance of different commercial kits using 3 IBVs antisera. Comparison of the mean S/P ratio of three IBV strains among three commercial IBV ELISA kits using Two-way ANOVA (a). Bubble plot of three different commercial kits (b). Pearson r value showing correlation of commercial ELISA kits of QX (c), M41 (d), and 793B (e) antisera using Pearson correlation coefficient. An asterisk denoted the significant levels (*), where ****, ***, ** and * indicate a p-value of < 0.0001, < 0.001, 0.01 and < 0.05, respectively.

None of the pathogen-free allantoic fluid inoculated groups showed positive results in commercial ELISA kits. The mean S/P ratios for the ELISA kit A showed two and one negative results out of 16 QX and 15 M41 antisera, respectively. In contrast, all 793B antisera were positive, with no significant difference in S/P ratios among the three antisera groups. Using the ELISA kit B, S/P ratios of each antisera group showed significant differences, with the 793B antisera exhibiting a significantly higher titre than the other two IBV antisera groups (Fig. 3a). The S/P ratio of all samples is illustrated in Fig. 3b. When using the ELISA kit C, samples in all antisera groups, including 11 of QX, two of M41, and four of 793B, showed negative results, particularly for QX antisera. Eleven samples of 16 QX antisera showed negative results on kits B. All 15 M41 and 793B antisera samples showed positive results on both ELISA kits (Table 1).

Using QX antisera, the ELISA kit A shows the highest sensitivity at 87.5%, followed by the ELISA kits B and C with 68.8% and 31.3%, respectively. For the M41 antisera group, all ELISA kits demonstrate sensitivity ≥ 86.7%. Similarly, for the 793B antisera group, the sensitivity of all ELISA kits ranges from 73.3% to 100% (Table 1). The Pearson correlation coefficient was used to establish correlations between commercial ELISA kits. The analysis showed a positive correlation: in assaying QX antisera, ELISA kit A was strongly correlated with ELISA kits B and C. In contrast, ELISA kits B and C were moderately correlated (Fig. 3c). Using M41 antisera, ELISA kit A was moderately correlated with ELISA kit C (Fig. 3d). Using 793B antisera, only ELISA kits A and C showed moderate correlation (Fig. 3e). The correlation between commercial ELISA kits was higher in the QX antisera groups (Fig. 3c) and lowest in the 793B antisera group (Fig. 3e).

There were relationships among ELISA kits A, B, and C when analysing all antiserum groups using simple linear regression. The proportion of the variance between ELISA kits A and B, A and C, and B and C showed positive relationships with R-squared values of 0.43, 0.34 and 0.11, respectively (P value < 0.05) (Table 2).

**Table 2:** Simple linear regression between commercial ELISA kits of all samples.

Simple linear regression			
R-squared	0.4332	0.3393	0.1117
p-value	<0.0001	<0.0001	0.0232
Equation	y = 2.78x + 0.1046	y = 0.2705x + 0.1307	y = 0.03671x + 0.2226

### Relationship analysis between HI titre and commercial ELISA result

Using Pearson correlation, the correlation analysis between homologous HI titres and commercial ELISA kit results reveals significant positive correlations for QX antisera with ELISA kits A, B, and C, with correlation coefficients of 0.53, 0.50 and 0.75, respectively. The correlations between other homologous HI tests and all ELISA kits are not statistically significant (Table 3).

**Table 3:** Correlation of homologous HI titre and commercial ELISA kits using Pearson.

Antisera	HI test	Correlation	ELISA kits		
			A	B	C
QX	QX HI	r	0.5256	0.4991	0.7463
		p-value	0.0365	0.0491	0.0009
M41	M41 HI	r	0.04783	−0.3367	0.4619
		p-value	0.8656	0.2198	0.083
793B	793B HI	r	−0.409	−0.1895	0.0728
		p-value	0.1301	0.4987	0.7965

**Fig. 4:**
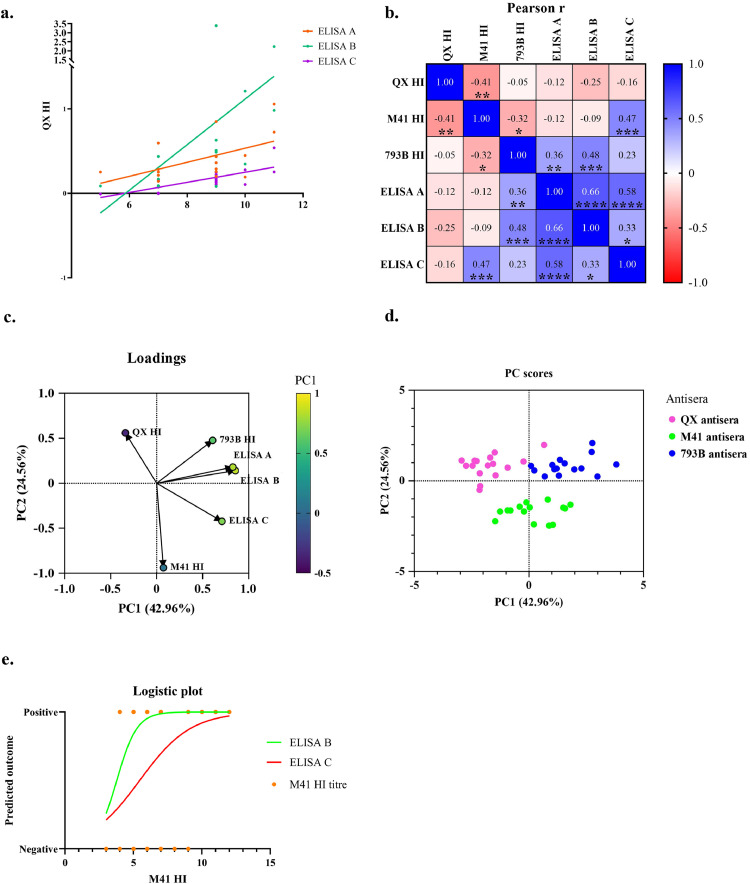
Relationship analysis between HI titre and commercial ELISA result. Scatterplots of the relationship between QX HI titre and S/P ratios of commercial ELISA kits of QX antisera (a). Pearson's r value of heterologous and homologous HI titre and S/P ratio of three commercial kits (b). Principal component analysis (PCA) loadings (c) and PCA scores (d) of serology test results of three IBV antisera groups. Logistic regression (e) of predicted positivity of ELISA kits B and C by M41 HI titre. An asterisk denoted the significant levels (*), where ****, ***, ** and * indicate a p-value of < 0.0001, < 0.001, 0.01 and < 0.05, respectively.

The scatterplots identified significant positive relationships between the QX HI titre and ELISA kit A, B, and C results (Fig. 4a). The R-squared values for the A, B, and C kits were 0.28, 0.25, and 0.56, respectively, in simple linear regression (p < 0.05). There is a significant positive correlation between the QX homologous HI titre, the A, B, and C commercial ELISA kits, and the M41 and 793B antisera. However, there are no statistically significant correlations between the homologous HI test and any of the commercial ELISA kits.

Relationships among all tests using the sera group were analysed using the Pearson correlation coefficient and Principal Component Analysis (PCA). Pearson's r values represent the correlation coefficients between the different tests. It demonstrated significant relationships as follows: QX HI titre exhibits a negative relationship with the M41 HI titre. The M41 HI titre shows a positive relationship with ELISA kits C and a negative relationship with the 793B HI titre. The 793B HI titre has positive correlations with ELISA kits A and B, and negative correlations with the M41 HI titre. All ELISA kit results showed positive correlations with one another but exhibited different relationships with the HI test. In particular, ELISA kits A and B showed a positive correlation with the 793B HI titre, and ELISA kit C demonstrated a positive relationship with the M41 HI titre, as shown in Fig. 4b. The PCA analysis reveals that a cumulative 67.52% of the total variance in the data is captured by PC1 and PC2, with 42.96% and 24.56%, respectively. PC1 is heavily influenced by the ELISA kits, with high positive loadings for ELISA kits A (0.75), B (0.76), and C (0.77), suggesting that PC1 represents the overall performance or response of the ELISA kits. Moderate loadings for 793B HI (0.55) and negative loadings for QX HI (−0.44) are also observed on PC1. PC2 shows moderate positive loadings for QX HI (0.48) and 793B HI (0.56), lower loadings for ELISA results of A (0.34) and B (0.35) ELISA kits, and high negative loadings for M41 HI (−0.90) and ELISA kits C (−0.26). PC2 differentiates between the QX HI and 793B HI titres, which have high positive loadings, and the M41 HI titres and ELISA kit C, which have high negative loadings. The loadings graph shows an arrow for three commercial ELISA kits pointing in a similar direction and demonstrating a strong correlation with PC1. At the same time, QX and M41 HI titres are separated, aligning with PC2, which captures their variability as illustrated in Fig. 4c.

The PCA score plot effectively visualises the distribution of the data points on the PC1 and PC2 axes, revealing three distinct clusters corresponding to the antisera groups: QX, M41, and 793B. QX antisera clusters are predominantly located in the PC2-positive regions and are highly influential in distinguishing QX antisera group from the others. Whereas all ELISA variables have high loadings on both PC1 and PC2, their influence on the QX antisera group is primarily through their contribution to PC2. The positioning of the M41 antisera clusters, mainly in the negative PC2 region, indicates different ELISA result patterns compared to the QX and 793B antisera groups. The high negative loadings of M41 HI on PC2 highlight its strong influence in separating the M41 antisera group, with M41 HI being a critical variable in defining this antisera group. In addition to M41 HI, the ELISA variables influence the separation of the M41 antisera group, but their impact is predominantly linked to PC1. The 793B antisera clusters are situated in the positive PC1 and PC2 regions. This result indicates that variables with positive loadings on both PC1 and PC2 are associated with high ELISA values and clearly distinguish themselves from QX and M41 antisera (Fig. 4d).

Logistic regression was conducted to identify the relationship between HI titre and the positivity of the ELISA kit result. The logistic regression analysis revealed that M41 HI titre was a significant predictor of kit B and C positivity, with odds ratios of 3.9 (95% CI: 1.5 to 18.2) and 1.7 (95% CI: 1.2 to 2.7), respectively (Fig. 4e). The model demonstrated good to excellent discriminative ability, with an area under the curve (AUC) of 0.9 (p-value = 0.0073), 0.8 (p-value = 0.0036), and 0.9 (p-value = 0.0177), respectively. Additionally, the Hosmer-Lemeshow test showed a satisfactory model fit. In comparison, no significant model with QX or 793B HI titres exists (Fig. 4e).

## Discussion

In this study, homologous HI reactions between antisera and haemagglutinating derived from the identical strain of IBV displayed maximum reactivity, with 100% sensitivity and high titres, and are ideal for detecting homologous strains. Whereas heterologous HI tests also show high sensitivity but pose some challenges, including the potential for false negatives and lower titres than those of the homologous HI assay. The reduction in titres of heterologous HI tests in this study highlights the decreased reactivity of heterologous antigens compared to homologous ones, as documented by King and Hopkins (King and Hopkins, 1983). PCA scores and loadings demonstrate separate clusters, exhibiting unique reactivity patterns. Moreover, the estimation of antigenic relationships among the three antisera groups was shown to be low. Our findings support the antigenic distinction among these three IBV strains. Similar to previous studies, the limited antigenic correlation of these strains suggests they belong to different serotypes (Yuan et al., 2023). Analysis in this study showed a significant positive relationship between QX HI and 793B HI titres, although these two strains have low antigenic relatedness (Xia et al., 2016). The different PCA loadings of heterologous and homologous HI tests on PC1 and PC2 highlight their distinct characteristics. This result indicates that heterologous HI tests exhibit varying degrees of cross-reactivity, suggesting some antigenic relationship between strains, but it is consistently lower than homologous interactions (Terreginol et al., 2008). Accompanied by the sensitivity results of the heterologous HI test in this study, suggesting that HI tests are ideal for serotype-specific monitoring. Similar to previous studies, HI tests involve multiple IBV strains and provide greater effectiveness than using only the M41 HI test (King, 1986). This is because cross-reactions among IBV strains occur due to similarities in antigenic epitopes, especially in conserved regions like the nucleocapsid (N) protein (Zhang et al., 2023), non-structural protein (nsP) (Lei et al., 2017), and S2 (Xie et al., 2023). Therefore, although heterologous HI tests can be performed effectively, challenges remain, particularly the risk of false negatives and reduced titres when vaccination or field exposure involves strains not included in the routine HI antigen panel, potentially leading to misdiagnosis. The present study utilised SPF chickens infected with a single IBV strain to establish defined serological profiles under controlled conditions. This approach was necessary to eliminate confounding factors and enable direct comparison of assay performance across distinct antigenic backgrounds. However, commercial poultry systems are considerably more complex, with birds commonly subjected to polyvalent vaccination programmes and exposure to heterologous field strains. Under such conditions, immune responses are shaped by prior antigenic exposure and may influence the hierarchy and specificity of antibody responses (Kim et al., 2012; Nait Mohamed and Lingwood, 2025). As a result, ELISA assays often reflect cumulative humoral immunity rather than strain-specific responses (Giacomo et al., 2025), while HI assays, although more indicative of serotype-related responses, remain affected by cross-reactivity (Lashgari and Newman, 1984b). In addition, coinfections or sequential infections with multiple IBV genotypes may further complicate serological interpretation (Bhuiyan et al., 2021). The findings of this study, including high heterologous HI sensitivity, reduced titres, and low antigenic relationship values, support the presence of partial antigenic overlap between strains used. Therefore, in field conditions, serological data should be interpreted alongside vaccination history, clinical findings, and molecular diagnostic methods such as PCR and sequencing. Our findings suggest that ELISA provides complementary insights into the immunological responses of different antisera. Moreover, ELISA kit performance varies depending on the kit and the antisera group. This result is consistent with Chen’s findings, which indicate that variability among ELISA kits indicates that some kits perform better with certain IBV strains (Chen et al., 2011). Such variability may be attributed to differences in IBV coating conditions used in the assay (Ahmed et al., 2007; Giardina et al., 2003). We found that some ELISA kits for QX antisera detection may not be sufficient due to varying effectiveness, especially for ELISA kits B and C. In contrast, ELISA kit A demonstrated strong ability to detect QX-specific antibodies. This finding has important implications for serological surveillance in IBV-negative flocks. When used alone, ELISA may produce false negative results. Therefore, ELISA could be complemented with other methods (such as western blot) to improve detection accuracy (Finger et al., 2018). In this study, the M41 antisera groups demonstrated consistently high sensitivity across all ELISA kits, indicating their strong effectiveness in detecting antibody responses to M41. This demonstrates a high reliability for monitoring this strain. Furthermore, the logistic regression analysis showed that the M41 HI results significantly predict ELISA outcomes. Though further studies are needed, results to date highlight that ELISA kit performance for M41 infection is linked to M41 HI titre levels. —Given that, it was found that the ELISA kits fail to provide accurate results in the flock with low M41 HI titre.

The 793B antisera exhibited the highest S/P ratios among all antisera groups across ELISA kits, aligning with earlier studies indicating that this strain elicits a robust antibody response when tested with the M41-coated ELISA kit (Benyeda et al., 2009). In this study, the three kits analysed showed variable effectiveness in detecting 793B-specific antibodies, highlighting the advantages and disadvantages of the kits. These findings underscore the importance of selecting an appropriate ELISA kit based on the dominant IBV strain prevalent in the flock, though some kits may detect multiple strains due to antigenic similarities. A recent study has reported an attempt to develop an ELISA kit to detect multiple IBV strains using conserved S2 peptides as the coating antigen, providing an effective tool for serological surveillance and vaccination monitoring (Xie et al., 2023). Our findings revealed significant differences among kits using the same serum group. This finding is supported by the WOAH Terrestrial Manual (World Organisation for Animal Health (WOAH), 2018), which states that the same serum sample may produce varying titres when tested with different IBV kits. The variability observed among commercial ELISA kits in this study is most likely attributable to differences in coating antigen composition. IBV ELISA systems may utilise whole-virus preparations, recombinant proteins, or specific antigenic components such as the nucleocapsid (N) protein, the spike S2 subunit, or the highly variable spike S1 subunit. The N protein (Lugovskaya et al., 2006) and S2 subunit (Xie et al., 2023) are relatively conserved and tend to promote cross-reactive antibody detection, whereas the S1 subunit is more antigenically diverse and associated with serotype-specific responses (Wang et al., 2002). Therefore, differences in antigen selection can substantially influence assay sensitivity and specificity across IBV strains (de Wit et al., 1997). The reduced sensitivity of certain ELISA kits for QX antisera, compared with the consistently high sensitivity of M41 antisera, likely reflects an antigenic mismatch between the coating antigen and the infecting strain. These findings are consistent with previous reports demonstrating that ELISA performance is influenced by antigen composition and coating conditions (Chen et al., 2011; Giardina et al., 2003), and that broader detection can be achieved using conserved antigenic targets such as S2 peptides (Xie et al., 2023). Importantly, these results indicate that antibody titres generated by different ELISA kits are not directly comparable and instead reflect the degree of antigenic alignment between the assay and circulating IBV strains.

In this study, a strong correlation was observed across all ELISA kits using Pearson correlation coefficients, suggesting they may measure similar underlying factors. These kits effectively capture the immune response despite different methodologies or antigen presentations.

PCA has been proposed as a valuable tool to support serological diagnostic testing in human medicine (Rymer et al., 1999; Schultz et al., 2024). In this study, we first analysed various IBV serological assays using specific antisera groups through PCA to explore patterns of antibody responses in chickens. According to score and loading plots, although the ELISA A, ELISA B and 793B HI loading vectors were not perfectly aligned, they all pointed toward the positive side of both PC1 and PC2, within the same quadrant where the 793B antisera group clustered. This finding suggests that for analysis of 793B sera, the ELISA kits of A and B and 793B HI may be the most informative tests for characterising the immune profile. In contrast, other ELISA kits appeared less effective at detecting antibodies in this antisera group, which is consistent with our performance analysis. These observations are supported by Kutle et al. (2020), who stated that immunisation with the 793B strain did not consistently result in seropositivity when assessed using an IDEXX IBV ELISA kit (Kutle et al., 2020). We found that M41 HI and ELISA C all load positively on PC1 and negatively on PC2; only M41 HI aligns closely with the region where the M41 antisera group clusters in the PC scores. These plots suggest that M41 HI reflects immune features characteristic of the M41 antisera group, making it the most representative test for this antisera group. This finding is consistent with previous studies reporting that serum from M41-vaccinated birds exhibited the highest HI titre when tested with the M41 antigen (Haa and Mag, 2015). The loading arrows for QX HI pointed toward the negative side of PC1 and the positive side of PC2, which coincides with the clustering of QX antisera on our loading plot. This close alignment indicates that QX HI is strongly related to the QX antisera and may serve as a functional assay to identify the antibody response in this antisera group. Supporting this interpretation, our performance analysis showed that QX HI is more sensitive than the other tests for assessing QX antisera. In this study, PCA proved to be a valuable exploratory tool for identifying serological tests associated with specific antisera groups and generating hypotheses on appropriate assays. However, PCA alone is insufficient for drawing definitive conclusions (Elhaik, 2022). The statistical and mathematical methods employed in this study facilitate data interpretation and reveal underlying patterns. Nonetheless, further targeted study is necessary to validate these exploratory findings.

Virus neutralisation (VN), also referred to as serum neutralisation (SN), is widely regarded as the reference assay for assessing functional immunity and differentiating IBV serotypes (Cook et al., 1987). However, VN was not included in the present study, as the primary objective was to compare routinely used serological assays under controlled experimental conditions rather than to validate a diagnostic method against a gold standard. In addition, IBV isolates require propagation in tracheal organ cultures (TOC) or embryonated chicken eggs (ECE) (Jiang et al., 2023), thereby introducing strain-dependent biological variability and limiting standardisation across isolates (Gharaibeh, 2007). Consequently, the implementation of VN across QX, M41 and 793B strains would have reduced inter-assay comparability. In contrast, HI and ELISA provide reproducible, standardised, and widely applicable methods for serological evaluation in both experimental and field settings. Homologous HI demonstrated 100% sensitivity across all antisera groups in this study, supporting its use as a consistent internal comparator. Nevertheless, the absence of VN should be considered a limitation, and future studies incorporating VN would further strengthen the interpretation of functional antibody responses.

Overall, this study provides a controlled framework for understanding assay-dependent variation in IBV serology, which is essential for interpreting more complex serological patterns observed under field conditions. This will enable effective disease management, including the selection of vaccine strains and vaccination strategies. For the diagnosis of IBV, this study suggests that using multiple assays, such as ELISA and HI, provides a greater understanding and interpretation of serological results. Findings from this study reinforce the advantages of using HI, as it profiles strain-specific IBV infections. We encourage concurrent use of both ELISA and HI, either for diagnosis or for monitoring responses after IBV vaccination, to improve flock health and production.

## Funding

This research was supported by HRH Princess Chulabhorn College of Medical Science, Chulabhorn Royal Academy, under “The Scholarship in Commemoration of Her Royal Highness Princess Chulabhorn’s 60th Birthday Anniversary”.

## CRediT authorship contribution statement

**Sirorat Munyahongse:** Writing – original draft, Validation, Resources, Methodology, Investigation, Formal analysis, Data curation. **Nicholas J. Evans:** Writing – review & editing, Supervision, Project administration. **Kannan Ganapathy:** Writing – review & editing, Supervision, Project administration, Methodology, Investigation, Funding acquisition, Conceptualization.

## Disclosures

The authors declare that they have no known financial, personal, or professional conflicts of interest that could have influenced the work reported in this manuscript. They also confirm that no competing interests exist that could be perceived as compromising the study's impartiality or integrity.

## References

[bib0001] Ahmed Z., Naeem K., Hameed A. (2007). Detection and seroprevalence of infectious bronchitis virus strains in commercial poultry in Pakistan. Poult. Sci..

[bib0002] Aldous E.W., K M.J., J B., Alexander D.J. (2003). A molecular epidemiological study of avian paramyxovirus type 1 (Newcastle disease virus) isolates by phylogenetic analysis of a partial nucleotide sequence of the fusion protein gene. Avian Pathol..

[bib0003] Alhajj M., Zubair M., Farhana A. (2023).

[bib0004] Beltrão N., Egochega R.F., Furian T.Q., Rodenbusch C.R., Fallavena L.C.B., Pasquali G., Canal C.W. (2012). A sensitive nested-polymerase chain reaction protocol to detect infectious laryngotracheitis virus. Acta Scientiae Veterinariae.

[bib0005] Benyeda Z., Mato T., Süveges T., Szabo E., Kardi V., Abonyi-Toth Z., Rusvai M., Palya V. (2009). Comparison of the pathogenicity of QX-like, M41 and 793/B infectious bronchitis strains from different pathological conditions. Avian Pathol..

[bib0006] Bhuiyan M.S.A., Amin Z., Bakar A., Saallah S., Yusuf N.H.M., Shaarani S.M., Siddiquee S. (2021). Factor influences for diagnosis and vaccination of avian infectious bronchitis virus (Gammacoronavirus) in chickens. J. Vet. Sci..

[bib0007] Bickerton E., Maier H.J., Stevenson-Leggett P., Armesto M., Britton P. (2018). The S2 subunit of infectious bronchitis virus beaudette is a determinant of cellular tropism. J. Virol..

[bib0008] Bradbury J.M. (1982). The use of chicken antiserum for the identification of avian mycoplasmas by immunofluorescence. Avian Pathol..

[bib0009] Cavanagh D., Mawditt K., Britton P., Naylor C. (1999). Longitudinal field studies of infectious bronchitis virus and avian pneumovirus in broilers using type-specific polymerase chain reactions. Avian Pathol..

[bib0010] Chen H.W., Wang C.H., Cheng I.C. (2011). A type-specific blocking ELISA for the detection of infectious bronchitis virus antibody. J. Virol. Methods.

[bib0011] Chhabra R., Forrester A., Lemiere S., Awad F., Chantrey J., Ganapathy K. (2015). Mucosal, cellular, and humoral immune responses induced by different live infectious bronchitis virus vaccination regimes and protection conferred against infectious Bronchitis Virus Q1 strain. Clin. Vaccine Immunol..

[bib0012] Comin A., Toft N., Stegeman A., Klinkenberg D., Marangon S. (2013). Serological diagnosis of avian influenza in poultry: is the haemagglutination inhibition test really the 'gold standard'?. Influenza Other Respir. Viruses..

[bib0013] Cook J.K., Brown A.J., Bracewell C.D. (1987). Comparison of the haemagglutination inhibition test and the serum neutralisation test in tracheal organ cultures for typing infectious bronchitis virus strains. Avian Pathol..

[bib0014] de Wit J.J., Malo A., Cook J.K.A (2019). Induction of IBV strain-specific neutralizing antibodies and broad spectrum protection in layer pullets primed with IBV Massachusetts (Mass) and 793B vaccines prior to injection of inactivated vaccine containing Mass antigen. Avian Pathol..

[bib0015] de Wit J.J., Mekkes D.R., Kouwenhoven B., Verheijden J.H. (1997). Sensitivity and specificity of serological tests for infectious bronchitis virus antibodies in broilers. Avian Pathol..

[bib0016] Elhaik E. (2022). Principal component analyses (PCA)-based findings in population genetic studies are highly biased and must be reevaluated. Sci. Rep..

[bib0017] Finger P.F., Pepe M.S., Dummer L.A., Magalhães C.G., de Castro C.C., de Oliveira Hübner S., Leite F.P.L., Ritterbusch G.A., Esteves P.A., Conceição F.R. (2018). Combined use of ELISA and western blot with recombinant N protein is a powerful tool for the immunodiagnosis of avian infectious bronchitis. Virol. J..

[bib0018] Gharaibeh S.M. (2007). Infectious bronchitis virus serotypes in poultry flocks in Jordan. Prev. Vet. Med..

[bib0019] Giacomo S.D., Geréz R., Olivera V., Asenzo G., Jatón J., Vagnozzi A.E. (2025). A standardized enzyme-linked immunosorbent assay (ELISA) for the detection of infectious Bronchitis virus antibodies in serum and tracheobronchial lavage samples of chickens. Avian Dis..

[bib0020] Giardina P.C., Evans R.E., Sikkema D.J., Madore D., Hildreth S.W. (2003). Effect of antigen coating conditions on enzyme-linked immunosorbent assay for detection of immunoglobulin G antibody to Neisseria meningitidis serogroup Y and W135 capsular polysaccharide antigens in serum. Clin. Diagn. Lab. Immunol..

[bib0021] Haa B., Mag H. (2015). Comparison between HI and ELISA in detecting immune titer following IBV vaccination. Assiut Veterin. Med. J..

[bib0022] Herlich H. (1965). Effect of Chicken antiserum and tissue extracts on the oocysts, sporozoites, and merozoites of eimeria tenella and E. acervulina. J. Parasitol..

[bib0023] Hitchner S. (1973). A virus neutralization screening test: its limitations in classifying field isolates of infectious bronchitis virus. Avian Pathol..

[bib0024] Ji P., Wang K., Zhang L., Yan Z., Kong M., Sun X., Zhang Q., Zhou N., Liu B., Zhou E.M., Sun Y., Wang X., Zhao Q. (2022). A new nanobody-enzyme fusion protein-linked immunoassay for detecting antibodies against influenza A virus in different species. J. Biol. Chem..

[bib0025] Jiang Y., Xue M., Tang M., Zhang D., Yu Y., Zhou S. (2023). Adaptation of the infectious bronchitis virus H120 vaccine strain to Vero cell lines. Vet. Microbiol..

[bib0026] Kim J.H., Davis W.G., Sambhara S., Jacob J. (2012). Strategies to alleviate original antigenic sin responses to influenza viruses. Proc. Natl. Acad. Sci. u S. a.

[bib0027] King D., Hopkins S. (1983). Evaluation of the hemagglutination-inhibition test for measuring the response of chickens to avian infectious bronchitis virus vaccination. Avian Dis..

[bib0028] King D.J. (1986). Serological profiles of commercial broiler breeders and their progeny. 1. Infectious bronchitis virus. Avian Dis..

[bib0029] Kutle L., Ljuma Skupnjak L., Vrdoljak A., Janković D., Boelm G.J., Kelemen F., Zorman Rojs O., Millecam J. (2020). Efficacy of infectious bronchitis GI-13 (793B) vaccine candidate tested according to the current European Union requirements and for cross-protection against heterologous QX-Like challenge. Viral. Immunol..

[bib0030] Lashgari M.S., Newman J.A. (1984). Determination of the antigenic relationships within the Massachusetts (M41) type of infectious bronchitis virus using the hemagglutination-inhibition test. Avian Dis..

[bib0031] Lashgari M.S., Newman J.A. (1984). Serological comparison and antigenic relationships of seven serotypes of infectious bronchitis virus using the hemagglutination-inhibition test. Avian Dis..

[bib0032] Lee C.-W., Hilt D.A., Jackwood M.W. (2001). Identification and analysis of the Georgia 98 serotype, a new serotype of infectious bronchitis virus. Avian Dis..

[bib0033] Lee C.W., Senne D.A., Suarez D.L. (2006). Development and application of reference antisera against 15 hemagglutinin subtypes of influenza virus by DNA vaccination of chickens. Clin. Vaccine Immunol..

[bib0034] Lei J., Shi T., Sun D., Mo K., Yan Y., Jin Y., Liao M., Zhou J. (2017). Development and application of nsp5-ELISA for the detection of antibody to infectious bronchitis virus. J. Virol. Methods.

[bib0035] Liebhart D., Bilic I., Grafl B., Hess C., Hess M. (2023). Diagnosing infectious diseases in poultry requires a holistic approach: a review. Poultry.

[bib0036] Lin S.Y., Chen H.W. (2017). Infectious Bronchitis virus variants: molecular analysis and pathogenicity investigation. Int. J. Mol. Sci..

[bib0037] Lugovskaya N.N., Scherbakov A.V., Yakovleva A.S., Tsyvanyuk M.A., Mudrak N.S., Drygin V.V., Borisov A.V. (2006). Detection of antibodies to avian infectious bronchitis virus by a recombinant nucleocapsid protein-based enzyme-linked immunosorbent assay. J. Virol. Methods.

[bib0038] Mao Q., Ma S., Schrickel P.L., Zhao P., Wang J., Zhang Y., Li S., Wang C. (2022). Review detection of Newcastle disease virus. Front. Vet. Sci..

[bib0039] Marquardt W., Snyder D., Schlotthober B. (1981). Detection and quantification of antibodies to infectious bronchitis virus by enzyme-linked immunosorbent assay. Avian Dis..

[bib0040] Mase M., Kanehira K. (2015). Surveillance of avian paramyxovirus serotype-1 in migratory waterfowls in Japan between 2011 and 2013. J. Vet. Sci..

[bib0041] Mohawk K.L., Melton-Celsa A.R., Robinson C.M., O'Brien A.D. (2010). Neutralizing antibodies to Shiga toxin type 2 (Stx2) reduce colonization of mice by Stx2-expressing Escherichia coli O157:H7. Vaccine.

[bib0042] Munoz J., Becker E.L. (1952). The use of chicken antiserum in the immunochemical studies of Edestin. J. Immunol..

[bib0043] Nait Mohamed F.A., Lingwood D. (2025). Innate immunity and training to subvert original antigenic sin by the humoral immune response. Elife.

[bib0044] Perrotta C., Furtek C., Wilson R.A., Cowen B.S., Eckroade R.J. (1988). A standardized enzyme-linked immunosorbent assay for infectious bronchitis virus: comparison with hemagglutination-inhibition and virus-neutralization assays for measuring protective antibody levels in chickens. Avian Dis..

[bib0045] Pillai-Kastoori L., Heaton S., Shiflett S.D., Roberts A.C., Solache A., Schutz-Geschwender A.R. (2020). Antibody validation for western blot: by the user, for the user. J. Biolog. Chem..

[bib0046] Rymer J.-C., Sabatier R., Daver A., Bourleaud J., Assicot M., Bremond J., Rapin J., Salhi S.L., Thirion B., Vassault A., Ingrand J., Pau B. (1999). A new approach for clinical biological assay comparison and standardization: application of principal component analysis to a multicenter study of twenty-one carcinoembryonic antigen immunoassay kits. Clin. Chem..

[bib0047] Schultz J.S., Okoli M., Lee S., Leonard C.M., Sayre D., Heilig C.M., Uhomoibhi P., Ogunniyi A., Ndodo N., Mba N., Abubakar A.G., Akinmulero O., Dawurung A.B., Okoye M., Iriemenam N.C., Plucinski M., Steinhardt L., Rogier E., Ihekweazu C. (2024). Principal component analysis of the serological response to plasmodium Falciparum using a Multiplex bead-based assay in Nigeria. Sci. Rep..

[bib0048] Sun J., Yan Y., Jiang J., Lu P. (2005). DNA immunization against very virulent infectious bursal disease virus with VP2-4-3 gene and chicken IL-6 gene. J. Veterin. Med., Ser. B.

[bib0049] Takase K., Murakawa Y., Ariyoshi R., Eriguchi S., Sugimura T., Fujikawa H. (2000). Serological monitoring on layer farms with specific pathogen-free chickens. J. Vet. Sci..

[bib0050] Terreginol C., Toffanl A., Beatol M.S., De Nardil R., Vascellail V., Meini A., Ortali G., Mancina M., Capual I. (2008). Pathogenicity of a QX strain of infectious bronchitis virus in specific pathogen free and commercial broiler chickens. Avian Pathol..

[bib0051] Villarreal L. (2010). Diagnosis of infectious bronchitis: an overview of concepts and tools. Brazil. J. Poultry Sci..

[bib0052] Wang C.H., Hong C.C., Seak J.C. (2002). An ELISA for antibodies against infectious bronchitis virus using an S1 spike polypeptide. Vet. Microbiol..

[bib0053] World Organisation for Animal Health (WOAH) (2018). Manual of Diagnostic Tests and Vaccines for Terrestrial Animals.

[bib0054] Xia J., He X., Yao K.-C., Du L.-J., Liu P., Yan Q.-G., Wen Y.-P., Cao S.-J., Han X.-F., Huang Y. (2016). Phylogenetic and antigenic analysis of avian infectious bronchitis virus in southwestern China, 2012–2016. Infect., Genet. Evolut..

[bib0055] Xie J., Meng X., Zhang J., Xie Q., Zhang W., Li T., Shao H., Wan Z., Qin A., Ye J. (2023). A novel S2-derived peptide-based ELISA for broad detection of antibody against infectious bronchitis virus. Poult. Sci..

[bib0056] Yuan W., Lv T., Jiang W., Hou Y., Wang Q., Ren J., Fan L., Xiang B., Lin Q., Ding C., Ren T., Chen L. (2023). Antigenic characterization of infectious bronchitis virus in the South China during 2021-2022. Viruses.

[bib0057] Zhang Y., Han Z., Li H., Liu S. (2023). Development of a recombinant enzyme-linked immunosorbent assay for the detection of antibodies against infectious Bronchitis virus. Viral. Immunol..

